# Genome-Wide Analysis of *CqCrRLK1L* and *CqRALF* Gene Families in *Chenopodium quinoa* and Their Roles in Salt Stress Response

**DOI:** 10.3389/fpls.2022.918594

**Published:** 2022-07-07

**Authors:** Wei Jiang, Chao Li, Leiting Li, Yali Li, Zhihao Wang, Feiyu Yu, Feng Yi, Jianhan Zhang, Jian-Kang Zhu, Heng Zhang, Yan Li, Chunzhao Zhao

**Affiliations:** ^1^National Key Laboratory of Crop Genetics and Germplasm Enhancement, National Center for Soybean Improvement, Key Laboratory for Biology and Genetic Improvement of Soybean (General, Ministry of Agriculture), Jiangsu Collaborative Innovation Center for Modern Crop Production, Nanjing Agricultural University, Nanjing, China; ^2^Shanghai Center for Plant Stress Biology, CAS Center for Excellence in Molecular Plant Sciences, Chinese Academy of Sciences, Shanghai, China; ^3^University of the Chinese Academy of Sciences, Beijing, China; ^4^The Bright Seed Industry Company, Shanghai, China; ^5^Agricultural Technology Center of Bright Rice (Group) Co., Ltd., Shanghai, China; ^6^National Key Laboratory of Plant Molecular Genetics, Shanghai Center for Plant Stress Biology, CAS Center for Excellence in Molecular Plant Sciences, Chinese Academy of Sciences, Shanghai, China

**Keywords:** *Chenopodium quinoa*, *CrRLK1Ls*, RALFs, salt stress, peptides

## Abstract

*Chenopodium quinoa* is a halophyte with exceptional nutritional qualities, and therefore it is potentially an ideal crop to grow in saline soils, not only addressing the problem of land salinization, but also providing nutrient food for the health of humans. Currently, the molecular mechanisms underlying salt tolerance in quinoa are still largely unknown. In *Arabidopsis thaliana*, *Catharanthus roseus* receptor-like kinase (*Cr*RLK1Ls) FERONIA (FER) and its ligands rapid alkalinization factors (RALFs) have been reported that participate in the regulation of salt tolerance. Here, we performed a genome-wide analysis and identified 26 *CqCrRLK1L* and 18 *CqRALF* family genes in quinoa genome. Transcriptomic profiling of the leaf, root, stamen, and pistil tissues of quinoa reveals that different *CqCrRLK1L* and *CqRALF* genes exhibit tissue-specific expression patterns, which is consistent with that observed in other plant species. RNA-seq data show that three *CqCrRLK1L* genes are highly up-regulated after salt treatment, suggesting that some *CqCrRLK1L* family genes are transcriptionally responsive to salt stress in quinoa. Biochemical study indicates that CqRALF15, a paralog of Arabidopsis RALF22, is physically associated with *Cr*RLK1L proteins CqFER and AtFER. CqRALF15 and AtRALF22 are functionally conserved in inducing the internalization of AtFER and in triggering root growth inhibition in both quinoa and Arabidopsis. Moreover, overexpression of *CqRALF15* in Arabidopsis results in enhanced leaf bleaching under salt stress, indicating that *CqRALF15* is involved in salt stress response. Together, our study characterizes *CqCrRLK1L* and *CqRALF* family genes in quinoa at genomic, transcriptional, and protein levels, and provides evidence to support their roles in salt stress response.

## Introduction

*Catharanthus roseus* receptor-like kinases (*Cr*RLK1Ls) are a family of receptor-like proteins that exist in many different plant species, ranging from charophytes to angiosperms (Zhu et al., 2021). The *Cr*RLK1L family proteins are characterized by two tandemly-linked malectin-like domains that are required for binding to cell wall polymers, such as pectin, and a kinase domain that replays apoplastic signals to intracellular components via a phosphorylation (Feng et al., 2018; Franck and Westermann, 2018; Lin et al., 2022). Because of the critical roles in sensing cell wall integrity, *Cr*RLK1L family proteins are required for the modulation of a wide range of biological processes, including plant growth, root hair elongation, fertility, immunity, and abiotic stress response (Duan et al., 2010; Haruta et al., 2014; Ge et al., 2017; Stegmann et al., 2017; Zhao et al., 2018). In Arabidopsis, there are 17 *Cr*RLK1L family proteins, and among of them the biological functions of FERONIA (FER), THESEUS1 (THE1), HERCULES1 (HERK1), ANXUR1/2 (ANX1/2), and BUDDHA’S PAPER SEAL1/2 (BUPS1/2) have been well studied. AtFER, which is universally expressed in both vegetative and reproductive tissues, is the most extensively studied *Cr*RLK1L family protein in Arabidopsis (Franck and Westermann, 2018). AtTHE1 was initially identified based on a mutant screen for suppressors that can rescue the short hypocotyl phenotype of cellulose-deficient mutant *procuste1-1* (*prc1-1*) (Hématy et al., 2007). It has also been reported that AtTHE1 and AtHERK1 function redundantly in regulating cell elongation (Guo et al., 2009), and AtFER, AtANJEA, and AtHERK1 form a heteromeric receptor complex to control polytubey block in Arabidopsis (Zhong et al., 2022). *AtANX1*/*2* and *AtBUPS1*/*2* are preferentially expressed in pollen tubes and participate in the regulation of pollen tube growth during fertilization (Miyazaki et al., 2009; Ge et al., 2017). Although *AtANX1*/*2* is weakly expressed in vegetative tissues, their roles in plant immunity in leaves have also been reported (Mang et al., 2017).

FER is named after an Etruscan goddess of fertility, because it was initially discovered as a critical regulator of pollen tube-ovule interaction (Huck et al., 2003; Rotman et al., 2003). During the last decade, many progresses have been achieved to decipher the novel functions of FER and the underlying molecular mechanisms. In Arabidopsis, FER regulates cell expansion, polarized cell growth, pathogen defense, and abiotic stress tolerance via the modulation of reactive oxygen species (ROS) balance (Huang et al., 2013) and the homeostasis of multiple phytohormones, such as jasmonic acid (JA), salicylic acid (SA), abscisic acid (ABA), brassinosteroid (BR), and ethylene (Guo et al., 2009, 2018; Deslauriers and Larsen, 2010; Zhao et al., 2021). Many intracellular components that are directly regulated by FER have been discovered, and one of the most well-known pathways is FER-GEFs (guanine exchange factors)-ROPs (Rho-GTPases of plants) signaling pathway. FER controls the activity of ROPs to regulate apoplastic ROS production, polarized cell elongation, and pavement cell morphogenesis (Duan et al., 2010; Huang et al., 2013; Lin et al., 2022; Tang et al., 2022). Recent studies indicate that FER participates in the regulation of salt tolerance, as mutation of *FER* results in enhanced leaf bleaching and short root elongation under high salinity (Feng et al., 2018; Zhao et al., 2018). It was pointed out that FER modulates cell wall integrity under salt stress via a Ca^2+^-mediated signaling pathway (Feng et al., 2018). Similar to *fer* mutation, *herk1* mutation combined with a gain of function allele of *the1* mutation also leads to leaf bleaching phenotype under salt stress (Gigli-Bisceglia et al., 2020). These results suggested that *Cr*RLK1L family proteins are required for salt tolerance in plants.

Based on biochemical, physiological, and genetic studies, it has been well demonstrated that rapid alkalinization factors (RALFs) are the ligands of *Cr*RLK1L family proteins (Haruta et al., 2014; Stegmann et al., 2017; Blackburn et al., 2020). In analogy to *CrRLK1L* family genes, different *RALFs* also exhibit tissue-specific expression patterns (Cao and Shi, 2012; Murphy and De Smet, 2014), which determines the tissue-specific pairs of *Cr*RLK1Ls and RALFs. For example, in Arabidopsis, AtFER recognizes AtRALF1, AtRALF22, and AtRALF23 that are dominantly expressed in leaves and roots (Haruta et al., 2014; Stegmann et al., 2017; Zhao et al., 2018). AtRALF4 and AtRALF19 are specifically expressed in pollen tubes, and thereby physically associate with pollen tube–specific AtANX1/2 and AtBUPS1/2 (Ge et al., 2017). A recent study showed that pollen tube–specific AtRALF6, 7, 16, 36, and 37 are recognized by AtFER, AtANJ, and AtHERK1 that are highly expressed in ovule (Zhong et al., 2022). In vegetative tissues, application of exogenous mature RALFs inhibits root growth in a process that depends on FER, and inhibition is proposed to be caused by the alkalization of apoplastic regions via the regulation of plasma membrane H^+^-ATPase AHA2 activity (Haruta et al., 2014; Abarca et al., 2021).

*Chenopodium quinoa* is an allotetraploid plant that originates from hybridization between diploid *Chenopodium pallidicaule* and diploid *Chenopodium suecicum* (Jarvis et al., 2017). Quinoa is a halophyte that processes a capacity to tolerate soil salinity, drought and cold stress, and sterile soil (Hinojosa et al., 2018). Due to its high nutritional value and stress tolerance property, quinoa has gained globally increased attention, and growth areas of quinoa have been dramatically increased in many countries during last few years. Whole-genome sequencing reveals that the genome size of quinoa is approximately 1.4 Gb and there are around 54,348 protein-encoding genes (Jarvis et al., 2017; Zou et al., 2017). Recently, quinoa has been selected as a model to study salt-tolerant mechanisms in halophyte (Zou et al., 2017). It is generally considered that epidermal bladder cells (EBCs) on the surface of quinoa are of primary importance for salt tolerance due to its capacity to accumulate high concentration of sodium in vacuoles (Kiani-Pouya et al., 2017; Böhm et al., 2018). However, a recent study reported that Na^+^ concentration is not substantially increased in the EBCs of salt-treated quinoa (Jaramillo Roman et al., 2020), suggesting that compensation mechanisms are associated with salt tolerance in quinoa. Therefore, identification of components required for salt tolerance would be critical for full understanding of salt-tolerant mechanisms in quinoa. In this study, we performed a genome-wide analysis of *CrRLK1L* and *RALF* family genes in quinoa and investigated the tissue-specific expression patterns of these two gene families and their roles in salt stress response.

## Materials and Methods

### Plant Materials and Growth Conditions

*Arabidopsis thaliana* wild-type (WT) was the Columbia (Col-0) ecotype. *fer-4* mutant and *AtFER-GFP* transgenic plants have been described previously (Zhao et al., 2018). The *C. quinoa* variety NL-6 was obtained from Prof. Heng Zhang. Both *Arabidopsis* and *C. quinoa* seeds were sterilized and sown on half Murashige and Skoog (MS) solid medium containing 1% sucrose and kept at 4°C for 2 days. Seeds were geminated in a light incubator at 22°C with a long-day cycle (16 h light/8h dark). For RNA-seq analysis, quinoa seedlings were transferred to soil and grown in a growth chamber at 22°C with a long-day cycle (16 h light/8 h dark).

### Plasmid Construction

For split luciferase complementation assay, the whole CDS sequences of *CqRALF15* and *AtRALF22*, as well as the ectodomain of *AtFER* and *CqFER*, were amplified by using Phanta^®^ Max Super-Fidelity DNA Polymerase (Vazyme, Nanjing, China). PCR products were purified by FastPure Gel DNA Extraction Mini Kit (Vazyme, Nanjing, China) and cloned into pDONR207 ENTRY vector using BP Clonase II Enzyme Mix (Thermo Fisher Scientific). Plasmids were extracted using FastPure Plasmid Mini Kit (Vazyme, Nanjing, China). Finally, all fragments mentioned above were recombined into pCAMBIA-nLUC or pCAMBIA-cLUC vectors using LR Clonase II Enzyme Mix (Thermo Fisher Scientific). For the generation of *CqRALF15* transgenic plants in Arabidopsis, *CqRALF15* was also recombined into the destination vector pEarleyGate 101. Primers used for constructs are listed in Supplementary Table 11.

### Mature RALF Peptides Treatment

For mature AtRALF22 and CqRALF15 treatment in Arabidopsis, WT and *fer-4* mutant seeds were geminated on 1/2 MS solid medium and grown for 5 d. Then the seedlings were transferred into 12-well plate. Each well of plate was filled with 4 mL of 1/2 Hoagland nutrient (pH 5.8) supplemented without or with 2 μM AtmRALF22 or CqmRALF15. The plates were gently shaken on a shaker in greenhouse. After treatment for 6 d, the root length of *Arabidopsis* seedlings was measured.

For mature RALF treatment in quinoa, the seeds of NL-6 were geminated on 1/2 MS solid medium for 2 days, and then seedlings were transferred into 5 mL centrifuge tube. Each centrifuge tube was filled with 4 mL of 1/2 Hoagland nutrient (pH 5.8) supplemented without or with 2 μM AtmRALF22 or CqmRALF15. The centrifugal tubes were placed in a light incubator. After treatment for 3 d, the root length of NL-6 seedlings was measured.

### Salt Stress Treatment

To conduct salt stress treatment, the seeds of wild type and *CqRALF15* transgenic plants were sown on 1/2 MS medium supplemented with or without 120 mM NaCl and kept at 4°C for 3 days before they were placed in an incubator at 22°C with long-day lighting conditions (16 h light/8 h dark). After growth for 12 days, the survival rate of seedlings on NaCl media was calculated.

### Identification of *CrRLK1L* and *RALF* Genes in Quinoa

The protein sequences of 17 CrRLK1L and 37 RALF genes in A. thaliana were downloaded from the Arabidopsis Information Resource (TAIR).^1^ The genomic databases of two sequenced quinoa varieties QQ74 and NL-6, were obtained from https://www.cbrc.kaust.edu.sa/chenopodiumdb/ and Prof.Heng Zhang, respectively.

To identify CrRLK1L genes in two quinoa genomes, Malectin_like (PF12819) and Pkinase_Tyr (PF07714) domains were downloaded from Pfam 35.0^2^ (Mistry et al., 2021). To identify CqRALF genes, RALF domain (PF05498) was downloaded. HMMER v3.0 (Finn et al., 2011) was used for searching quinoa sequences with the conserved domains, and SMART^3^ (Letunic and Bork, 2018) was used to manually check the domain architectures of candidate proteins. In addition, using the Arabidopsis and quinoa protein sequences as queries, sequence alingment and phylogenetic analysis were performed, and the protein sequences lacking conserved domains or conserved residues were removed.

### Chromosomal Locations, Phylogenetic Analysis of the *CrRLK1L* and *RALF* Genes

The physical locations of *CrRLK1L* genes on chromosomes were analyzed based on QQ74 genome database (Jarvis et al., 2017) and drawn by MG2C^4^ (Chao et al., 2021).

*Cr*RLK1L or RALF protein sequences of quinoa and Arabidopsis were aligned using Muscle 3 (Edgar, 2004). The phylogenetic trees were constructed by using IQ-TREE2 with the best-fit model and 1,000 replicates of ultrafast bootstrap (Minh et al., 2020). The resulting tree files were submitted to iTOL (Letunic and Bork, 2021) for modification.

### Protein Domain Structure of Cq*Cr*RLK1Ls and CqRALFs

TBtools (Chen et al., 2020) was used to display Cq*Cr*RLK1L protein domains that were predicted by SMART. Multiple sequence alignments of CqRALFs were subjected to CLC Sequence Viewer 8^5^ for visualization and the conserved residues and motifs of CqRALFs were indicated.

### RNA-Seq Analysis

NL-6 variety was used for transcriptomic analysis. For root sample, NL-6 were sown on half MS medium, and roots were collected after growth under dark conditions for three days. Stamen and pistil samples were collected from newly opened flowers of quinoa after growth on soils for about seven weeks. Vegetative leaves were sampled from 14-day-old seedlings either exposed to 400 mM NaCl for 8 h or under normal conditions. These different tissue samples were immediately frozen in liquid nitrogen and stored at –80°C.

Total RNA extraction, mRNA purification, library preparation, Illumina NovaSeq 6000 paired-end (150 bp) sequencing, transcriptomic assembly and analysis were all performed by Personalbio (Shanghai, China). The clean reads that were filtered from raw reads were aligned to the genome reference of quinoa NL-6 variety using HISAT2 (Kim et al., 2019). The number of read hits for each gene was calculated by HTSeq (Anders et al., 2015), and then converted into FPKM (fragments per kilobase of transcript per million reads mapped). Differential expression analysis was performed using DESeq based on the criteria: p ≤ 0.05 and | log_2_(fold change)| ≥ 1. Heatmaps were generated by TBtools based on the row Z-Scores of FPKM values of *CqCrRLK1L* and *CqRALF* genes.

### RNA Extraction and Quantitative RT–PCR

Total RNAs of all samples were extracted by using EastepTM Super Total RNA Extraction Kit (Promega, United States). cDNAs were synthesized using HiScript III RT SuperMix for qPCR (+gDNA wiper) according to the manufacturer’s instructions (Vazyme, Nanjing, China). qRT-PCR was performed using ChamQ Universal SYBR qPCR Master Mix (Vazyme, Nanjing, China) and Bio-Rad CFX connect real time detection system (BIO-RAD, United States). Primers used for qRT-PCR are listed in Supplementary Table 11.

### Split Luciferase Complementation Assay

*Agrobacterium* (GV3101 strain) harboring different recombinant plasmids were harvested from liquid Lysogeny Broth (LB) by centrifugation. Each strain was suspended in 10 mM MgCl_2_ with a final concentration of OD_600_ = 0.8–1.0. Then two different strains carrying indicated nLUC- and cLUC-tagged constructs were mixed with an equal volume. The mixed strains were added with 200 μM acetosyringone and 10 mM MES (pH 5.6) and incubated at room temperature for 3 h. The mixed strains were infiltrated into the leaves of *N. benthamiana* using a 1 mL disposable syringe. After 48 h, D-Luciferin was injected into the same tobacco leaves and the fluorescence was visualized by living plant imaging system (LB985 NightSHADE, Germany) after 10 min.

### Confocal Imaging

*AtFER-GFP* transgenic seeds were germinated and grown on 1/2 MS solid medium for 5 days, and then the seedlings were transferred into 1/2 liquid MS medium. After incubation overnight, the seedlings were treated with 2 μM AtmRALF22 or 2 μM CqmRALF22 peptide for 1 h before the roots were subjected to confocal imaging. GFP fluorescence was detected by Leica confocal laser scanning microscope SP8 with 488 nm excitation light and 510–550 nm emission light.

## Results

### Identification and Characterization of *Cr*RLK1L Family Proteins in Quinoa

**FIGURE 1:**
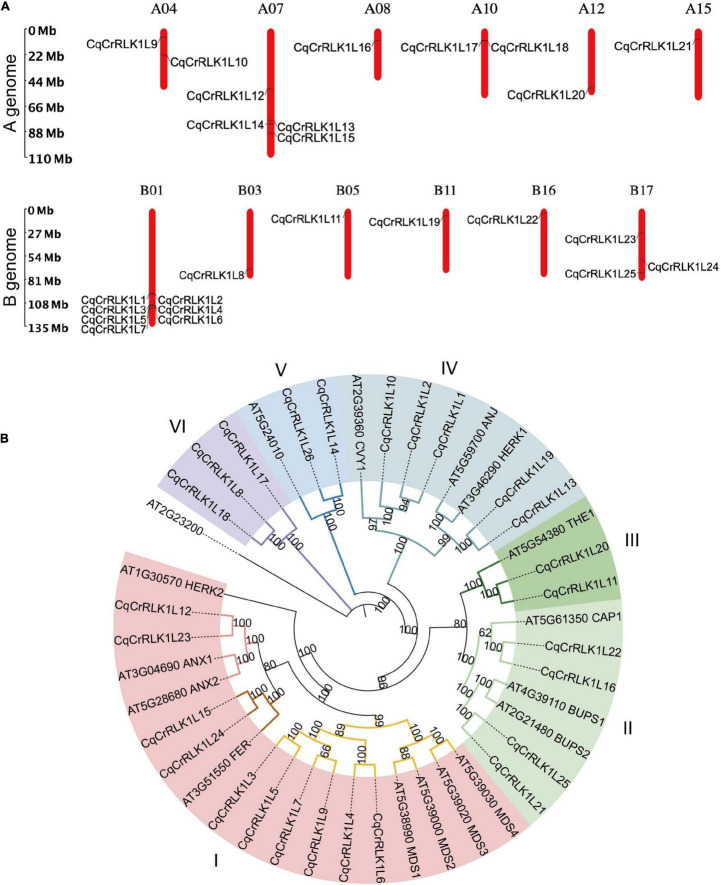
Identification and characterization of *CqCrRLK1L* genes in *Chenopodium quinoa*. **(A)** Distribution of *CqCrRLK1L* genes on the chromosomes of quinoa is shown. The chromosomes of A and B genomes are presented separately. **(B)** Phylogenetic analysis of *Cr*RLK1L proteins in quinoa and Arabidopsis. The full-length amino acids of *Cr*RLK1L proteins were aligned and phylogenetic tree was generated by using IQ-TREE2 program.

To identify *Cr*RLK1L family proteins in quinoa, we aligned the sequences of all 17 Arabidopsis *Cr*RLK1Ls proteins against the protein database of quinoa. Because quinoa QQ74 variety genome is currently the most well annotated genome (Jarvis et al., 2017), we explored this genome as a reference in this study. Combining the information of sequence similarity and the canonical malectin and kinase domains of C*r*RLK1L family proteins, totally 26 *Cr*RLK1L-like proteins were identified in quinoa, which were designed as Cq*Cr*RLK1L1 to Cq*Cr*RLK1L26 based on their locations on chromosomes, and these proteins were all predicted to be localized at plasma membrane (Supplementary Table 1). Quinoa genome consists of 18 chromosomes, and 25 *CqCrRLK1L* genes have been annotated on these chromosomes, while only *CqCrRLK1L26* has not been mapped yet (Figure 1A). Interestingly, *CqCrRLK1L3*-*CqCrRLK1L6* were tandemly localized on chr1, and protein sequences alignment showed that Cq*Cr*RLK1L3 and Cq*Cr*RLK1L5 share 100% protein identity, while Cq*Cr*RLK1L4 and Cq*Cr*RLK1L6 share an identity of 89.2%, implying a tandem duplication event occurred in this region. Besides, Cq*Cr*RLK1L1 and Cq*Cr*RLK1L2 as well as Cq*Cr*RLK1L17 and Cq*Cr*RLK1L18 are closely located on chr1 and chr10, respectively (Figure 1A). *Cr*RLK1L family proteins are characterized by their malectin domain and kinase domain. We performed protein structural analysis and found that all 26 CqC*r*RLK1L proteins harbor a canonical extracellular malectin-like domain and a cytosolic kinase domain (Supplementary Figure 1).

To understand the evolutionary relationship of these Cq*Cr*RLK1L proteins with their equivalents in Arabidopsis, phylogenetic analysis was performed (Figure 1B). The *Cr*RLK1L proteins were classified into six subgroups based on homology between Arabidopsis and quinoa, and for most of *Cr*RLK1L proteins in Arabidopsis, their counterparts were identified in quinoa (Figure 1B). In Arabidopsis, several C*r*RLK1L proteins, including AtFER, AtTHE1, AtCAP1, AtANX1/2, AtBUPS1/2, AtHERK1, AtANJ, and AtCVY1, have been well characterized. In quinoa, two copies of CqFER (Cq*Cr*RLK1L15/Cq*Cr*RLK1L24), CqTHE1 (Cq*Cr*RLK1L11/Cq*Cr*RLK1L20), CqCAP1 (Cq*Cr*RLK1L16/Cq*Cr*RLK1L22), CqANX1/2 (Cq*Cr*RLK1L12/Cq*Cr*RLK1L23), CqBUPS1/2 (Cq*Cr*RLK1L21/Cq*Cr*RLK1L25), and CqHERK1/ANJ (Cq*Cr*RLK1L13/Cq*Cr*RLK1L19), and three copies of CqCVY1 (Cq*Cr*RLK1L1/Cq*Cr*RLK1L2/Cq*Cr*RLK1L10) were identified (Figure 1B). Notably, for each of these duplicates they were identified on A genome and B genome of quinoa, respectively (Figure 1A), suggesting that they originated from the two ancestors of quinoa.

### Tissue-Specific Expression Patterns of *CqCrRLK1L* Genes

In Arabidopsis, *CrRLK1L* genes are expressed in a tissue-specific pattern. For example, *AtFER* is ubiquitously expressed in both vegetative and reproductive tissues, while *AtANX1/2* and *AtBUPS1/2* are dominantly expressed in pollen tubes (Franck and Westermann, 2018). To understand whether *CqCrRLK1Ls* genes in quinoa also exhibit tissue-specific expression patterns, we collected leaf, root, stamen, and pistil tissues of quinoa NL-6 variety, and performed RNA-seq analysis. Three independent replicates were conducted for each tissue. Based on the criterion that the reads matched to genes should be detected in all three independent replicates, totally 32,368, 35,204, 31,257, and 36,068 genes were expressed in leaves, roots, stamen, and pistil, respectively. Combining all these four samples, totally 40,456 genes were detected in our RNA-seq data (Supplementary Table 2), accounting for approximately 69% of predicted genes in quinoa. Among these genes, 462 were specifically expressed in leaf, 1935 were specifically expressed in root, 901 were specifically expressed in stamen, and 1098 were specifically expressed in pistil (Supplementary Table 3).

**FIGURE 2:**
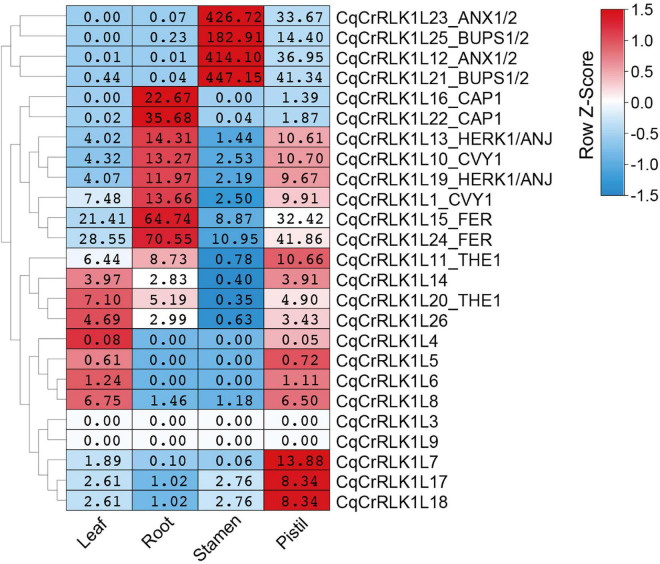
Tissue-specific expression pattern of *CqCrRLK1L* genes in quinoa. RNA-seq analysis was performed for the leaf, root, stamen, and pistil tissues of quinoa, and three independent replicates were conducted for each tissue. For each replicate, 8 quinoa roots were collected from three-day-old seedlings grown on half MS medium under dark conditions; more than 80 stamens and 30 pistils were sampled from newly opened flowers after growth on soils for seven weeks; all vegetative leaves were collected from four independent 14-day-old seedlings. The expression of each *CqCrRLK1L* gene in all four tissues was analyzed based on RNA-seq data. For each *CqCrRLK1L* gene, its expression in four tissues was normalized by row scale before plotting. Different colors in the heatmap indicate the relative expression level of each gene in different tissues based on the row Z-scores of the fragments per kilobase of transcript per million reads mapped (FPKM), while the digital numbers presented in the heatmap cells indicate the accurate values of the averaged FPKM of the three independent biological replicates.

By using these RNA-seq data, we analyzed the expression of all *CqCrRLK1L* genes. For the 26 *CqCrRLK1L* genes in quinoa QQ74 variety, 25 of them were identified in quinoa NL-6 variety (Supplementary Table 1). RNA-seq data showed that the expressions of 23 *CqCrRLK1L* genes were detected in all four or specific tissues, while two genes *CqCrRLK1L3* and *CqCrRLK1L9* were not or weakly detected in all four RNA-seq samples (Supplementary Table 4). We then analyzed the expression patterns of the 23 *CqCrRLK1L* genes in different tissues. *CqAUX1/2* and *CqBUPS1/2* were dominantly expressed in stamen, while *CqCrRLK1L7*, *CqCrRLK1L17*, and *CqCrRLK1L18* were mainly expressed in pistil. *CqACAP1* was mainly detected in root, and *CqHERK1* and *CqCURVY1* were relatively highly expressed in root and pistil. *CqTHE1*, *CrRLK1L14*, and *CrRLK1L26* were evenly expressed in leaf, root, and pistil. Similar to *AtFER* in Arabidopsis, *CqFER* was ubiquitously expressed in all four tissues (Figure 2). Remarkably, two copies of *CqFER*, *CqANX1, CqBUPS1/2*, *CqHERK1*, *CqCAP1*, *CqCVY1*, and *CqTHE1* exhibited very similar expression patterns (Figure 2), corroborating that they are the duplicated genes originating from the two ancestors of quinoa. Collectively, these results suggested that *CqCrRLK1L* genes in quinoa also exhibit tissue-specific expression patterns, and the expression patterns are reminiscent of their paralogs in Arabidopsis.

### Transcriptional Analysis of *CqCrRLK1L* Genes in Response to Salt Stress

Studies have shown that AtFER, AtTHE1, and AtHERK1 are involved in the regulation of salt tolerance in Arabidopsis (Zhao et al., 2018; Gigli-Bisceglia et al., 2020). To decipher whether salt stress affects the expression of *CqCrRLK1L* genes in quinoa, we performed RNA-seq analysis for the leaves of quinoa NL-6 variety before and after salt treatment. Totally three independent biological replicates were performed. RNA-seq data revealed that 2,772 genes were significantly up-regulated (fold change > 2, *p* value < 0.05), while 3096 genes were significantly down-regulated after salt treatment (fold change > 2, *p* value < 0.05) (Supplementary Table 5).

**FIGURE 3:**
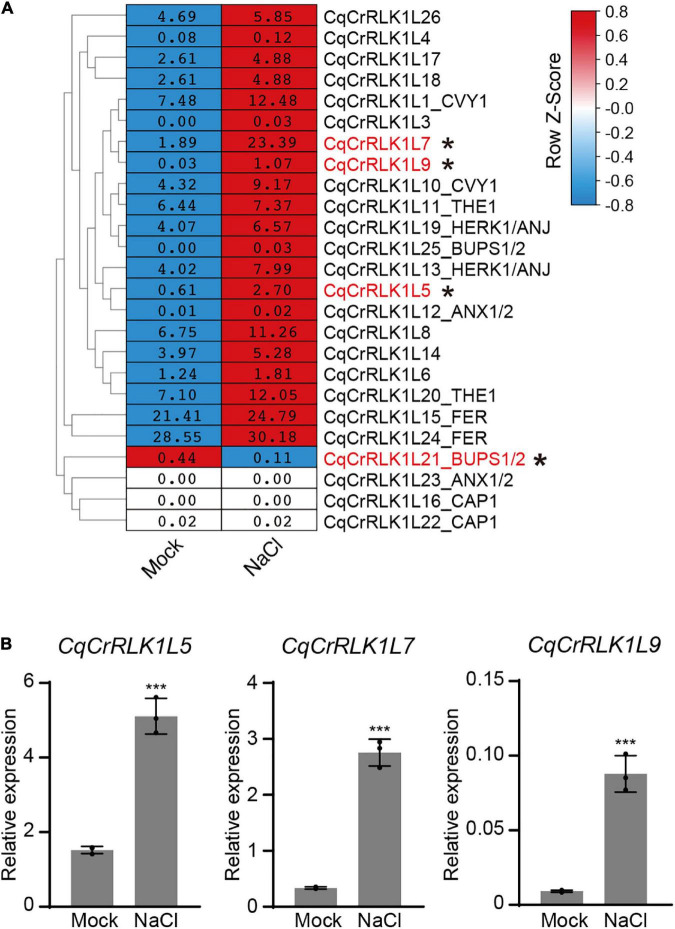
Analysis of the expression of *CqCrRLK1L* genes in response to salt stress. **(A)** RNA-seq analysis was performed for the leaves of quinoa before and after NaCl treatment. 14-day-old seedlings were treated with or without 400 mM NaCl for 8 h, and for each replicate all vegetative leaves from four different plants of the same treatment were collected and totally three replicates were conducted. The mean FPKM value of each *CqCrRLK1L* gene was normalized by row scale before plotting. Different colors in the heatmap indicate the relative expression level of each gene before and after NaCl treatment based on the row Z-scores of FPKM, while the digital numbers presented in the heatmap cells represent the averaged FPKM of the three independent biological replicates. Differential expression analysis was performed using DESeq, and the *CqCrRLK1L* genes that were significantly up-regulated or down-regulated (*p* ≤ 0.05 and | log_2_(fold change)| ≥ 1) are marked by asterisks. **(B)** Quantitative real-time (qRT)-PCR analysis of *CqCrRLK1L5*, *CqCrRLK1L7*, and *CqCrRLK1L9* genes before and after NaCl (400 mM) treatment. qRT-qPCR assay was performed using samples independent of RNA-seq samples. *CqEF1a* was used as an internal control. Values are the means ± SD of three biological replicates. Asterisks indicate statistically significant differences (***p < 0.001, Student’s *t*-test).

Based on RNA-seq data, we found that the expression of *CqFER*, *CqTHE1*, and *CqHERK1* genes was not significantly changed after NaCl treatment based on the criterion of fold change > 2, (Figure 3A and Supplementary Table 6), which is consistent with that reported in Arabidopsis (Zhao et al., 2021). These results suggested that the responses of these three *CrRLK1L* genes to salt stress were not regulated at a transcriptional level. Nevertheless, RNA-seq data showed that the expression of 21 *CqCrRLK1L* genes was increased after salt stress, and among of them three *CqCrRLK1L* genes, *CqCrRLK1L5*, *CqCrRLK1L7*, and *CqCrRLK1L9*, were significantly up-regulated after salt treatment (fold change > 2, *p* value < 0.05). Specifically, *CqCrRLK1L9* was increased more than 30-fold, *CqCrRLK1L7* was increased around 10-old, and *CqCrRLK1L5* was increased 3.7-fold (Figure 3A and Supplementary Table 6). qRT-PCR analysis verified that the transcript levels of *CqCrRLK1L5*, *CqCrRLK1L7*, and *CqCrRLK1L9* were dramatically increased after salt treatment (Figure 3B). Intriguingly, these three genes are grouped in a same cluster in the phylogenetic tree and the close homologs of these genes were not identified in Arabidopsis (Figure 1B), implying that these three genes may function redundantly and uniquely in quinoa to regulate salt stress response. Our RNA-seq data showed that, although *CqCrRLK1L21*_*BUPS1/2* was dominantly expressed in reproductive tissues, its expression was also weekly detected in leaves, and salt stress treatment significantly repressed its expression (fold change > 2, *p* value < 0.05) (Figure 3A and Supplementary Table 6). Independent qRT-PCR was also performed to validate the expression of *CqCrRLK1L21*_*BUPS1/2* gene before and after salt stress, but due to its extremely low expression level, the expression of this gene in leaves could not be effectively detected by using qRT-PCR assay.

### Tissue-Specific Expression Patterns of *CqRALFs* in Quinoa

It has been experimentally demonstrated that RALFs are the ligands of *Cr*RLK1L family proteins, and there are approximately 37 RALFs in Arabidopsis (Abarca et al., 2021). By aligning all these AtRALFs against the database of both quinoa QQ74 and NL-6 varieties, 18 paralogs were identified and these genes were named as *CqRALF1*-*CqRALF18* based on their locations on chromosomes (Supplementary Table 7). Notably, *CqRALF3* and *CqRALF4*, as well as *CqRALF15* and *CqRALF16*, are tandemly arrayed on quinoa chromosomes, and the genomic sequences of all these four genes were identified in the genome of both QQ74 and NL-6 varieties. However, in the latter genome, both *CqRALF3*/*CqRALF4* and *CqRALF15*/*CqRALF16* pairs were annotated as one gene (Supplementary Table 7), and they need to be manually corrected in the new version of genome annotation.

**FIGURE 4:**
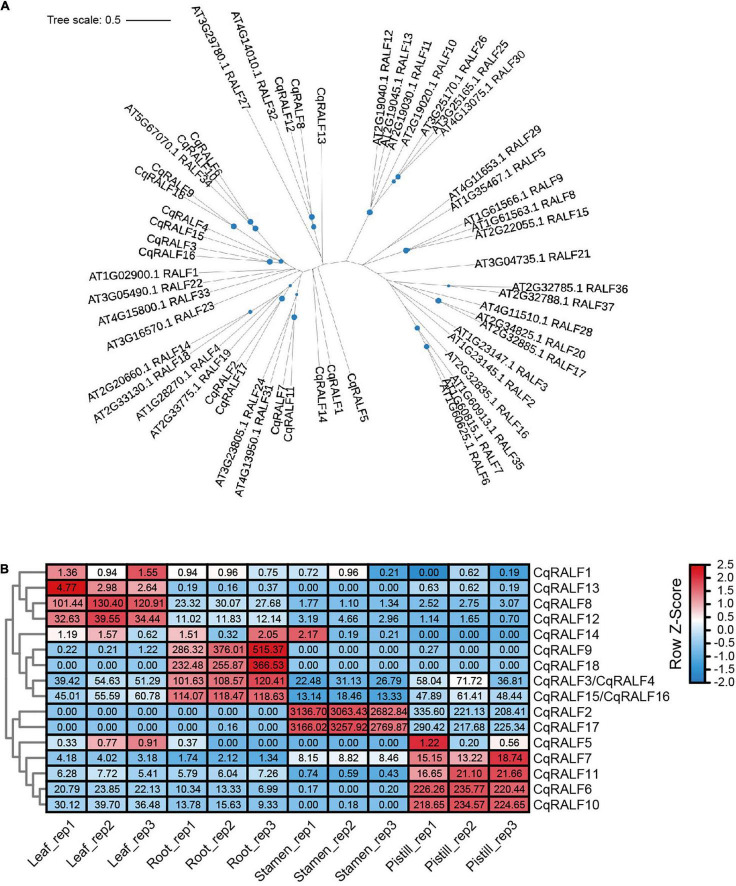
Characterization of *CqRALFs* in quinoa. **(A)** Phylogenetic analysis of RALFs in quinoa and Arabidopsis. The full-length amino acids of RALF peptides were used for phylogenetic analysis, and phylogenetic tree was generated by using IQ-TREE2 program. **(B)** Analysis of the tissue-specific expression patterns of *CqRALFs* in leaf, root, stamen, and pistil tissues of quinoa. The RNA-seq data used for gene expression analysis are the same as that described in Figure 2. The FPKM values of each *CqRALF* gene in four tissues were normalized by row scale before plotting. Different colors in the heatmap indicate the relative expression level of each gene in different tissues based on the row Z-scores of FPKM, while the digital numbers presented in the heatmap cells represent the accurate FPKM values in three independent biological replicates. Notably, *CqRALF3*/*CqRALF4* and *CqRALF15*/*CqRALF16* pairs were annotated as one gene in the current version of NL-6 genome annotation.

In analogy to AtRALFs, the typical features of RRXL motif, YISY motif, and four conserved cysteines were also identified in all 18 CqRALF peptides (Supplementary Figure 2), corroborating that these motifs are evolutionarily conserved and are critical for RALFs to conduct their functions. Phylogenetic analysis between quinoa and Arabidopsis showed that the well-studied AtRALF1, AtRALF22, and AtRALF23 peptides in Arabidopsis were closely associated with CqRALF3, CqRALF4, CqRALF15, and CqRALF16, while AtRALF4 and AtRALF19, which are specifically expressed in pollen tubes and participates in the regulation of pollen tube growth in Arabidopsis (Ge et al., 2017), were closely linked to CqRALF2 and CqRALF17 (Figure 4A).

Transcriptomic analysis showed that all these *CqRALF* genes were detected in all or either of the four tissues (Supplementary Table 8). Specifically, *CqRALF2* and *CqRALF17* were extremely highly expressed in stamen, and less expressed in pistil, but their transcripts were not detected at all in leaves and roots (Figure 4B and Supplementary Table 8). This expression pattern was reminiscent of their paralogs *AtRALF4* and *AtRALF19* in Arabidopsis (Ge et al., 2017), suggesting that these two *CqRALFs* may specifically participate in the regulation of reproduction process. *CqRALF6* and *CqRALF10* were highly expressed in pistil, but not expressed in stamen. *CqRALF8* and *CqRALF12* were dominantly expressed in leaves and roots, and weakly expressed in stamens and pistils (Figure 4B and Supplementary Table 8). Interestingly, *CqRALF9* and *CqRALF18* were only expressed in roots, but not expressed in other tissues (Figure 4B and Supplementary Table 8), suggesting that they may play a pivotal role in the regulation of root development. These results indicated that, similar to the *RALFs* in other plant species, *RALFs* in quinoa also exhibited obvious tissue-specific expression patterns. We also analyzed the expression of these *CqRALF* genes in response to salt stress, and none of them were significantly affected after salt treatment (fold change > 2, *p* value < 0.05) (Supplementary Table 9).

### CqRALF15 Is Physically Associated With CqFER and AtFER

**FIGURE 5:**
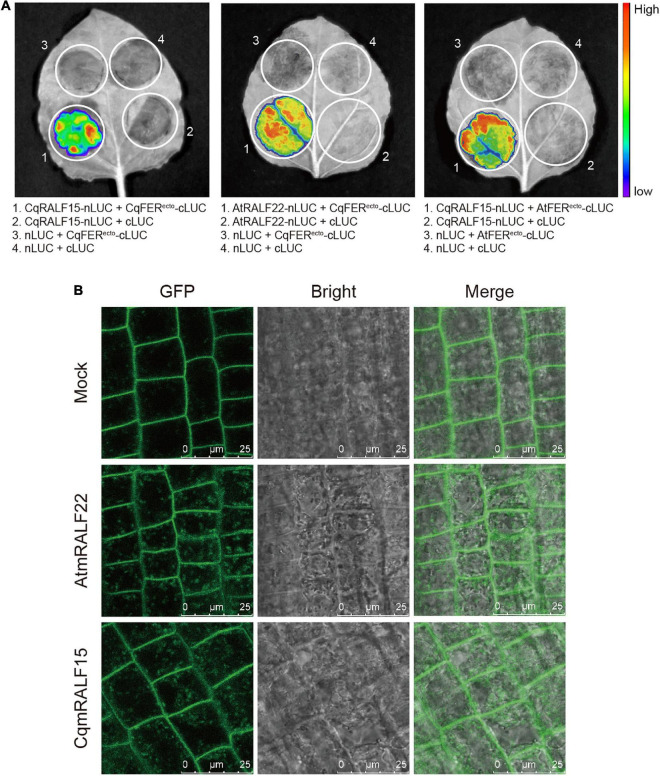
CqRALF15 interacts with both CqFER and AtFER. **(A)** Split luciferase complementation assays showing the interactions of CqRALF15 with the ectodomain of CqFER (CqFER^ecto^) and AtFER (AtFER^ecto^), as well as the interaction of AtRALF22 with CqFER^ecto^. Fluorescence was detected at 48 h after infiltration of the indicated constructs. **(B)** Analysis of the internalization of FER-GFP after mature RALF peptides treatment. Six-day-old seedlings were treated without or with 2 μM CqmRALF15 or AtmRALF22 for 1 h. Fluorescence in root cells were detected using confocal laser scanning microscopy. Bar = 25 μm.

AtRALFs are physically associated with At*Cr*RLK1L proteins in Arabidopsis (Stegmann et al., 2017; Zhao et al., 2018). To test whether this is also the case in quinoa, we chose CqRALF15, a CqRALF with the best hit after aligning AtRALF22 with quinoa genome, and analyze its interaction with CqFER. Split-LUC assay indicated that CqRALF15 interacts with the ectodomain of CqFER (CqFER^ecto^) (Figure 5A), which verified the formation of RALF-FER complex in plants. Besides, split-LUC assay indicated that AtRALF22 from Arabidopsis also interacts with CqFER^ecto^, and CqRALF15 interacts with AtFER^ecto^ (Figure 5A), indicating that RALFs and FER from quinoa and Arabidopsis are conserved in their physical interactions.

Studies have shown that treatment of *AtFER-GFP* transgenic plants with exogenous mature AtRALFs triggers the internalization of AtFER that colocalizes with the endocytic tracer FM4-64 in Arabidopsis (Zhao et al., 2018; Yu et al., 2020). Here, to further clarify the functional conservation of RALFs in Arabidopsis and quinoa, we synthesized mature AtRALF22 (AtmRALF22) and mature CqRALF15 (CqmRALF15) peptides (Supplementary Table 10). Under normal conditions, AtFER-GFP fusion protein was dominantly localized at plasma membrane. Application of AtmRALF22, however, rapidly triggered the internalization of FER-GFP (Figure 5B). Similarly, treatment of seedlings with CqmRALF15 was also able to trigger the internalization of FER-GFP (Figure 5B), verifying that AtRALF22 and CqRALF15 are functionally conserved.

### CqmRALF15 and AtmRALF22 Inhibits Root Growth in Both Quinoa and Arabidopsis

**FIGURE 6:**
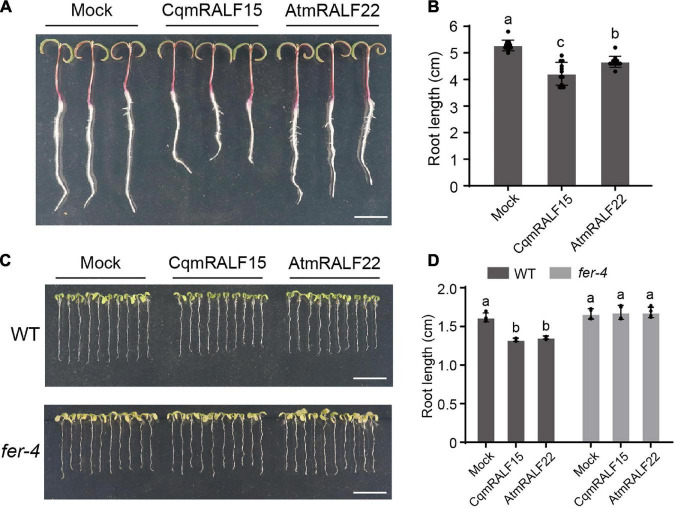
CqmRALF15 and AtmRALF22 inhibit the root growth of both quinoa and Arabidopsis. **(A)** Two-day-old seedlings of quinoa NL-6 treated with 2 μM CqmRALF15 or AtmRALF22. The picture was photographed after treatment for 3 days. Bar = 1 cm. **(B)** Measurement of the root length of quinoa seedlings shown in panel **(A)**. Values are the means ± SD (*n* = 12 seedlings). Different letters indicate statistically significant differences (*p* < 0.01, one-way ANOVA). **(C)** Arabidopsis wild type seedlings treated with CqmRALF15 or AtmRALF22. Five-day-old wild type and *fer-4* seedlings grown in liquid medium overnight were treated with 2 μM CqmRALF15 or AtmRALF22. After treatment for 6 days, the seedlings were placed on solid MS medium for photograph. Bar = 1 cm. **(D)** Quantification of the root length of seedlings shown in panel **(C)**. Values are the means of four independent replicates ± SD (n = 10–15 for each replicate). Different letters indicate statistically significant differences (*p* < 0.01, one-way ANOVA).

RALFs are well characterized by their ability to inhibit root growth (Haruta et al., 2014), so we tested the influence of mature RALF peptides on the root growth of quinoa. Our results showed that both CqmRALF15 and AtmRALF22 inhibited root elongation in quinoa (Figures 6A,B). Notably, with a same concentration, CqmRALF15 had a more serious effect than AtmRALF22 on root growth inhibition in quinoa (Figures 6A,B). We also treated Arabidopsis seedlings with CqmRALF15 and AtmRALF22, and found that these two mature RALFs exhibited a comparable effect on root growth inhibition in Arabidopsis (Figures 6C,D). It is known that AtRALF1-mediated root growth inhibition depends on its receptor AtFER (Haruta et al., 2014). Here both CqmRALF15- and AtmRALF22-triggered root growth inhibition was abolished in *fer-4* mutant (Figures 6C,D), indicating that CqmRALF15 and AtmRALF22 exhibit a similar biological function via FER-mediated signaling pathways.

### Overexpression of *CqRALF15* in Arabidopsis Leads to Leaf Bleaching Phenotype Under High Salinity

In Arabidopsis, FER was shown to be involved in salt tolerance (Feng et al., 2018; Zhao et al., 2018), so we were interested in elucidating whether CqFER in quinoa is also required for salt tolerance. Because genetic transformation in quinoa is still technically challenging, we attempted to transform *CqFER* construct to *fer-4* mutant in Arabidopsis and test whether *CqFER* can complement the leaf bleaching phenotype of *fer-4* mutant under salt stress. However, we failed to generate *CqFER* transgenic construct, because this construct was unstable in *Escherichia coli* strain DH5α grown under different growth conditions. More than 12 independent strain clones were selected for plasmid isolation and Sanger sequencing, but only *CqFER* constructs with missense/non-sense mutations or truncated fragments were obtained. We speculated that the instability of *CqFER* construct is because of the constitutive kinase activity of CqFER, which may be detrimental to *E. coli*. This assumption was supported by the fact that correct FER construct was successfully obtained when K551, an ATP binding residue that is essential for the kinase activity of CqFER, was substituted with alanine.

**FIGURE 7:**
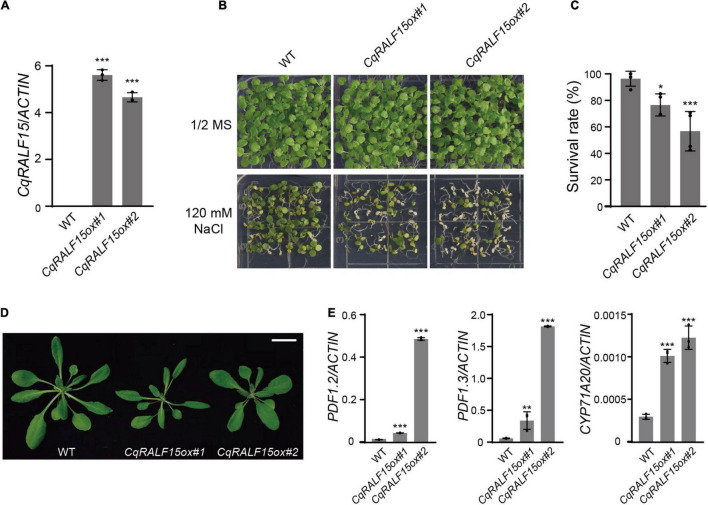
Arabidopsis transgenic plants overexpressing *CqRALF22* exhibit enhanced leaf bleaching under salt stress. **(A)** qRT-PCR analysis of *CqRALF22* transcript level in *CqRALF22* transgenic plants in Arabidopsis. *ACTIN* gene was used as an internal control. Values are the means ± SD of three biological replicates. Asterisks indicate statistically significant differences (***p < 0.001, Student’s *t*-est). **(B)** Phenotype of 12-day-old seedlings grown on 1/2 MS medium supplemented without or with NaCl (120 mM). **(C)** Survival rate of seedlings grown on NaCl medium (120 mM). The seedlings with full leaf bleaching were considered as dead plants. Values are the means ± SD of four biological replicates (*n* = 42 seedlings). Asterisks indicate statistically significant differences (*p < 0.05, ***p < 0.001, Student’s *t*-test). **(D)** Phenotype of each genotype grown in soil for 25 days. Bar = 2 cm. **(E)** Analysis of the expression of *PDF1.2*, *PDF1.3*, and *CYP71A20* genes in *CqRALF22*-overexpressing plants. Values are the means ± SD of three biological replicates. Asterisks indicate statistically significant differences (**p < 0.01, ***p < 0.001, Student’s *t*-test).

Similar to *fer-4* mutant, Arabidopsis plants overexpressing *RALF22* or *RALF23* exhibit leaf bleaching phenotype under salt stress (Zhao et al., 2018). We then generated *CqRALF15* construct driven by 35S promoter and transformed this construct to wild type Arabidopsis plants. qRT-PCR analysis showed that *CqRALF15* was highly expressed in the transgenic plants (Figure 7A). Phenotype analysis showed that overexpression of *CqRALF15* in Arabidopsis results in enhanced leaf bleaching phenotype under high salinity (Figures 7B,C), indicating that *CqRALF15* in quinoa also participates in the regulation of salt tolerance. In addition, it has been reported that Arabidopsis plants overexpressing *AtRALF22* or *AtRALF23* exhibit dwarf phenotype, which is caused by the over-activation of JA signaling pathway (Guo et al., 2018; Zhao et al., 2021). Here, *CqRALF15*-overexpressing plants exhibited a smaller plant size and higher expression levels of JA-responsive genes, such as *PDF1.2*, *PDF1.3*, and *CYP71A20*, than the wild type plants (Figures 7D,E), suggesting that *CqRALF15* is also involved in the regulation of plant growth and JA signaling pathway.

## Discussion

With increasing efforts on the study of *CrRLK1L* and *RALF* family genes, the importance of these genes in the regulation of plant development and stress responses has been highlighted in the past decade. Therefore, identification and characterization of *CrRLK1L* and *RALF* family genes in different plant species will advance our understanding of the roles of *CrRLK1L* and *RALF* genes at both evolutionary and plant species-specific levels. In this study, we performed a genome-wide analysis for *CrRLK1L* and *RALF* family genes in quinoa and identified 26 *CqCrRLK1L* and 18 *CqRALF* genes, respectively. The number of *CrRLK1L* genes in quinoa is more than that in Arabidopsis, which is because of the allotetraploid property of quinoa that contains duplicated genes originating from two ancestors. Compared with approximately 37 *RALF* genes in Arabidopsis (Abarca et al., 2021), the amount of *RALF* genes in quinoa is much less, which could be due to evolutionary divergence or different terrestrial habitats. Because current quinoa genome assembly and annotations have not been fully completed yet, we still cannot exclude the possibility that some *CqRALF* genes were missing in our analysis.

*Cr*RLK1L family proteins share similar protein structural domains, but different family members exhibit distinct biological functions, which is to some extent caused by the tissue-specific expression patterns of these *CrRLK1L* genes (Franck and Westermann, 2018). One of the best examples in Arabidopsis is that *AtBUPS1* and *AtBUPS2* are specifically expressed in pollen tube and they are required for pollen tube elongation (Ge et al., 2017). In quinoa, *CqBUPS1/2* was dominantly expressed in stamen, suggesting that *CqBUPS1/2* probably also contribute to the regulation of pollen tube growth in quinoa. Currently, FER is considered as one of the most important *Cr*RLK1L family proteins, because it participates in multiple biological processes, including cell expansion, root hair growth, fertility, plant immunity, and abiotic stress response (Duan et al., 2010; Haruta et al., 2014; Stegmann et al., 2017; Zhao et al., 2018; Huang et al., 2020; Ortiz-Morea et al., 2020; Zhong et al., 2022). These biological functions coincide with the ubiquitous expression of *AtFER* in leaf, primary root, root hair, and ovule in Arabidopsis (Franck and Westermann, 2018). RNA-seq data showed that *CqFER* was also highly expressed in leaf, root, and pistil in quinoa, indicating that *CqFER* probably exhibits similar biological functions as *AtFER*. Together, these results not only elucidate the tissue-specific expression patterns of *CrRLK1L* family members in quinoa, but also reveal that the expression patterns of *CrRLK1L* genes are conserved in different plant species. Similarly, *RALF* genes in quinoa also exhibited an obvious tissue-specific expression pattern, suggesting that different *CqRALFs* may execute distinct biological functions depending on the tissues they are expressed. For those *CqRALF* genes that are specifically expressed in reproductive tissues or in roots, their roles in fertility and root development merits further investigations. Besides, which Cq*Cr*RLK1Ls and CqRALFs are coupled in quinoa to regulate a specific physiological process requires further exploration.

Transcriptome profiling indicated that the transcript levels of *FER*, *THE1*, and *HERK1*, which are required for salt tolerance in plants (Zhao et al., 2018; Gigli-Bisceglia et al., 2020), are not significantly up-regulated after salt treatment in both quinoa and Arabidopsis (Zhao et al., 2021), suggesting that these three proteins may undergo posttranslational modifications in response to salt stress. It has been reported that ABA, a major phytohormone responsive to abiotic stress, can trigger the phosphorylation of FER (Chen et al., 2016). Therefore, whether these three *Cr*RLK1L proteins undergo phosphorylation modifications upon exposure to salt stress needs to be addressed in future. Interestingly, RNA-seq data revealed that the expression of three phylogenetically clustered *CqCrRLK1L* genes in quinoa was highly up-regulated after salt treatment, indicating that partial *CqCrRLK1L* genes are transcriptionally regulated in response to salt stress. Because the close paralogs of these three *CqCrRLK1L* genes do not exist in Arabidopsis, it will be quite interesting to investigate whether they uniquely evolved in quinoa and confer quinoa to tolerate high concentration of salts.

*RALFs* are identified in a wide range of land plants but not in charophytes, while *CrRLK1Ls* are found in both land plants and charophytes, suggesting that *RALFs* probably emerged later than *CrRLK1Ls* during evolution (Zhu et al., 2021). Evolutionary study indicates that *RALFs* experienced rapid expansion after separation of eudicot and monocot species, and the number of *RALFs* varies greatly among different plant species, implying that *RALFs* appeared accompanied by plant adaptation to different terrestrial habitats (Cao and Shi, 2012). Although the number of *RALFs* in quinoa is less than that in Arabidopsis, *CqRALF15* and its Arabidopsis paralog AtRALF22 exhibited similar functions, as both of them interacted with FER protein and triggered the internalization of AtFER. Besides, in analogy to mature *AtRALF22*, application of mature *CqRALF15* inhibited the root growth of both quinoa and Arabidopsis. These results suggest that RALF paralogs in quinoa and Arabidopsis are functionally conserved. In Arabidopsis, overexpression of *AtRALF22* or *AtRALF23* leads to pronounced leaf bleaching under salt stress (Zhao et al., 2018), and here the Arabidopsis plants overexpressing *CqRALF15* also exhibited enhanced leaf bleaching under salt stress, indicating that *CqRALF15* processes a similar function as *AtRALF22*/*23* in the regulation of salt tolerance. In future, whether other *CqRALFs* also participate in salt stress response needs to be further studied.

Quinoa is a natural halophyte, and the mechanisms underlying its salt tolerance have gained increasing attention, but to date salt tolerance mechanisms in quinoa still lack molecular insights. Elucidation of the roles of Cq*Cr*RLK1Ls and CqRALFs in the regulation of salt tolerance will guide us to take a close view of the functional specificity of Cq*Cr*RLK1Ls and CqRALFs in quinoa and lay a foundation for mechanical insights of salt tolerance in quinoa. EBCs are the typical feature that confers salt tolerance in quinoa. In future, the expression of *CqCrRLK1Ls* and *CqRALFs* in EBCs after salt treatment needs to be investigated, especially for those genes that are required for the regulation of salt stress response.

## Data Availability Statement

The datasets presented in this study can be found in online repositories. The RNA-seq data have been deposited in the NCBI GEO under accession number: GSE198572.

## Author Contributions

WJ, CL, and CZ contributed to conception and design of the study. WJ and CL performed the most of experiments. YLL and ZW assisted the growth of quinoa. LL and HZ conducted genome assembly and annotation of quinoa NL-6. FYu, FYi, and JZ provided quinoa materials. HZ and J-KZ participated in scientific discussions. YL and CZ supervised the research. CZ wrote the manuscript with the contributions of WJ and CL. All authors contributed to the article and approved the submitted version.

## Conflict of Interest

FYu was employed by Agricultural Technology Center of Bright Rice (group) Co., Ltd., Shanghai, China and FYi and JZ were employed by The Bright Seed Industry Company, Shanghai, China. The remaining authors declare that the research was conducted in the absence of any commercial or financial relationships that could be construed as a potential conflict of interest.

## Publisher’s Note

All claims expressed in this article are solely those of the authors and do not necessarily represent those of their affiliated organizations, or those of the publisher, the editors and the reviewers. Any product that may be evaluated in this article, or claim that may be made by its manufacturer, is not guaranteed or endorsed by the publisher.

## Footnotes

1
http://www.arabidopsis.org

2
http://pfam.xfam.org

3
http://smart.embl-heidelberg.de

4
http://mg2c.iask.in/mg2c_v2.1/

5
https://digitalinsights.qiagen.com/products/clc-sequence-viewer-direct-download/

## Supplementary Material

Supplementary Figure 1 Protein structural analysis of Cq*Cr*RLK1L proteins. Predicted domains in each Cq*Cr*RLK1L protein are shown. Signal peptide, malectin-like domain, transmembrane domain, and kinase domain are marked as dark green, green, yellow, and red, respectively.

Supplementary Figure 2 Protein structural analysis of CqRALF peptides. The full-length amino acids of all 18 CqRALF peptides were aligned, and the motifs RRXL, YISY, and four conserved cysteines are indicated.
