# Supplementary material for: Genome-Wide Analysis of CqCrRLK1L and CqRALF Gene Families in Chenopodium quinoa and Their Roles in Salt Stress Response
Source: Front Plant Sci. 2022 Jul 7;13:918594. doi: 10.3389/fpls.2022.918594 (PMC9302450; doi:10.3389/fpls.2022.918594)
Supplement: Supplementary file 10 [file Table_10.DOCX]

**Supplementary Table 10. Peptide sequences used in this study.**

| **Peptide name** | **Sequence** |
| --- | --- |
| AtmRALF22 | AQKKYISYGAMRRNSVPCSRRGASYYNCQRGAQANPYSRGCSTITRCRR |
| CqmRALF15 | ATNNYISYNALNKNRVPCSRRGASYYNCRPGAQANPYSRGCSRITRCARR |
