# Supplementary material for: Genome-Wide Analysis of CqCrRLK1L and CqRALF Gene Families in Chenopodium quinoa and Their Roles in Salt Stress Response
Source: Front Plant Sci. 2022 Jul 7;13:918594. doi: 10.3389/fpls.2022.918594 (PMC9302450; doi:10.3389/fpls.2022.918594)
Supplement: Supplementary file 11 [file Table_11.DOCX]

**Supplementary Table 11. Primers used in this study.**

| **Primer name** | **Sequence** | **Usage** |
| --- | --- | --- |
| fer-4 LP | GGAAAATGAGAGAACAGAGAACAA | Genotyping |
| fer-4 RP | CTTCTGTGAGTTCCTTGTCTCTCTC | Genotyping |
| LBa (GABI) | ATATTGACCATCATACTCATTGC | Genotyping |
| PDF1.3 RT-LP | CTAAGTCTGCTGCCATCATCACT | Quantitative real-time PCR |
| PDF1.3 RT-RP | ATGTTTTGCCCCCTCAAGGT | Quantitative real-time PCR |
| ACTIN RT-LP | ATGACTCAGATCATGTTTGAGACC | Quantitative real-time PCR |
| ACTIN RT-RP | TCAGTAAGGTCACGACCAGCAA | Quantitative real-time PCR |
| CqCrRLK1L5 RT-LP | GGTGGGGGTCAAATCACCAT | Quantitative real-time PCR |
| CqCrRLK1L5 RT-RP | GATTGTCGAGGTTGGTGGGT | Quantitative real-time PCR |
| CqCrRLK1L7 RT-LP | CCTGCGGTATCTCCAAGTCC | Quantitative real-time PCR |
| CqCrRLK1L7 RT-RP | GCGGTGGTGAAATAAAGGCG | Quantitative real-time PCR |
| CqCrRLK1L9 RT-LP | GGGAGTGCAGGGTACCTAGA | Quantitative real-time PCR |
| CqCrRLK1L9 RT-RP | TGGCCCATGAGATTTGGGTC | Quantitative real-time PCR |
| CqRALF15 RT-LP | TGGGGTTATTCGGAAGTGGAT | Quantitative real-time PCR |
| CqRALF15 RT-RP | ATACGACTGCAACCACGAGA | Quantitative real-time PCR |
| CqEF1a RT-LP | CAATGGTATGGCTGAGGGTCC | Quantitative real-time PCR |
| CqEF1a RT-RP | CAGGTAGCATCCACTTCTCAGG | Quantitative real-time PCR |
| pDONR207-AtRALF22 LP | GGGGACAAGTTTGTACAAAAAAGCAGGCTTCACCATGACGAACACTCGCGCG | Plasmid construction for split-LUC assay |
| pDONR207-AtRALF22 RP | GGGGACCACTTTGTACAAGAAAGCTGGGTCACGCCTGCACCTAGTGATGG | Plasmid construction for split-LUC assay |
| pDONR207-ectoAtFER LP | GGGGACAAGTTTGTACAAAAAAGCAGGCTTCACCATGGCTGATTACTCTCCAACAGAGA | Plasmid construction for split-LUC assay |
| pDONR207-ectoAtFER RP | GGGGACCACTTTGTACAAGAAAGCTGGGTCAGCCGTATTGCTTTTCGATTTC | Plasmid construction for split-LUC assay |
| pDONR207-CqRALF15 LP | GGGGACAAGTTTGTACAAAAAAGCAGGCTTCACCATGGCCGACAATAGAAAGCTAG | Plasmid construction for split-LUC assay and transgenic plants |
| pDONR207-CqRALF15 RP | GGGGACCACTTTGTACAAGAAAGCTGGGTCGCGTCTTGCACAACGAGTG | Plasmid construction for split-LUC assay and transgenic plants |
| pDONR207-ectoCqFER LP | GGGGACAAGTTTGTACAAAAAAGCAGGCTTCACCATGGGGAGAAAATTAGCTTTG | Plasmid construction for split-LUC assay |
| pDONR207-ectoCqFER RP | GGGGACCACTTTGTACAAGAAAGCTGGGTCAACAACCTCCTGCGTTGG | Plasmid construction for split-LUC assay |
