# Supplementary figures and images for: Genome-Wide Analysis of CqCrRLK1L and CqRALF Gene Families in Chenopodium quinoa and Their Roles in Salt Stress Response
Source: Front Plant Sci. 2022 Jul 7;13:918594. doi: 10.3389/fpls.2022.918594 (PMC9302450; doi:10.3389/fpls.2022.918594)

Figure S1

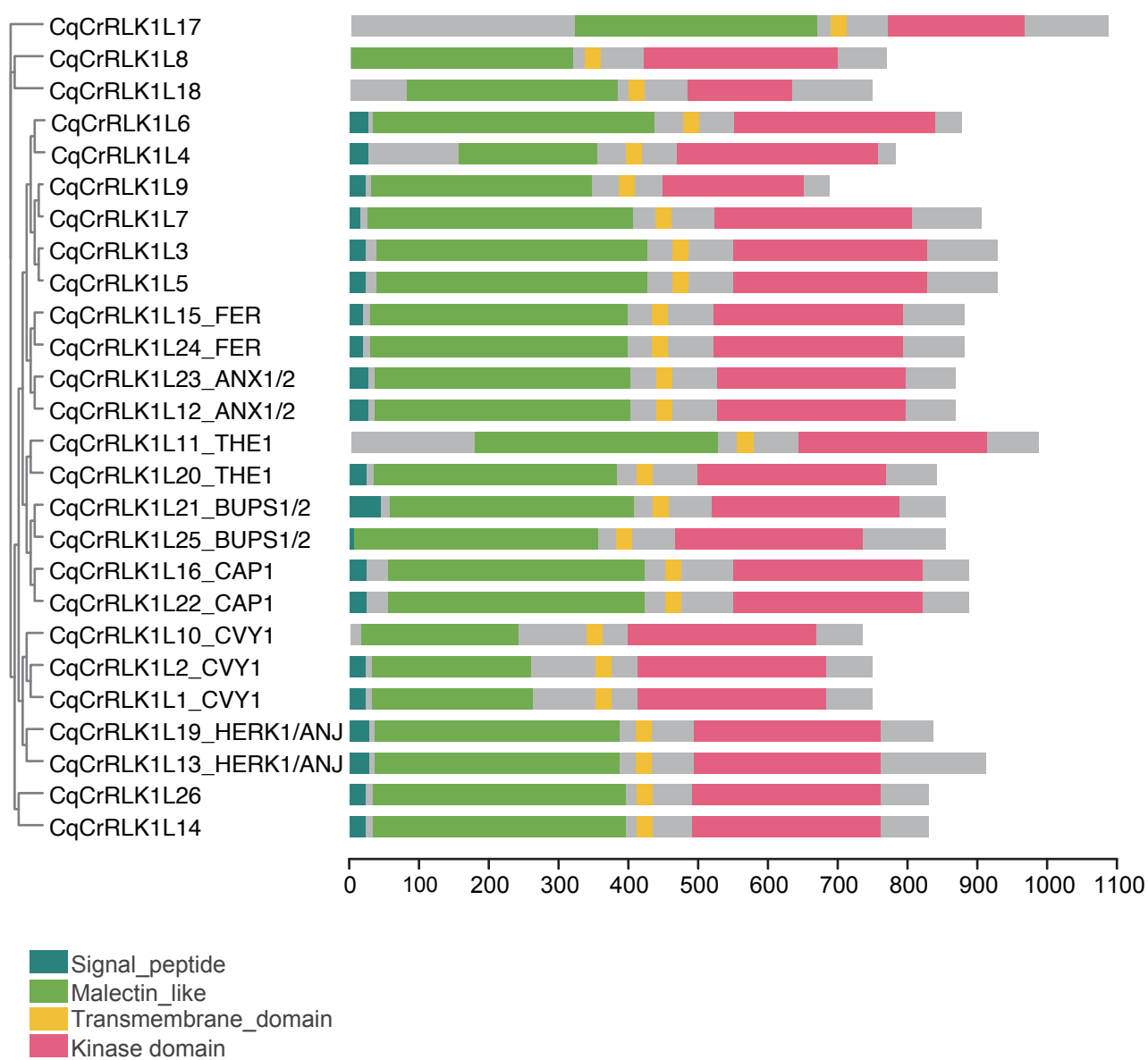

Supplement: Supplementary Figure 1 — Protein structural analysis of CqCrRLK1L proteins. Predicted domains in each CqCrRLK1L protein are shown. Signal peptide, malectin-like domain, transmembrane domain, and kinase domain are marked as dark green, green, yellow, and red, respectively. [file Data_Sheet_1.PDF]

Figure S2

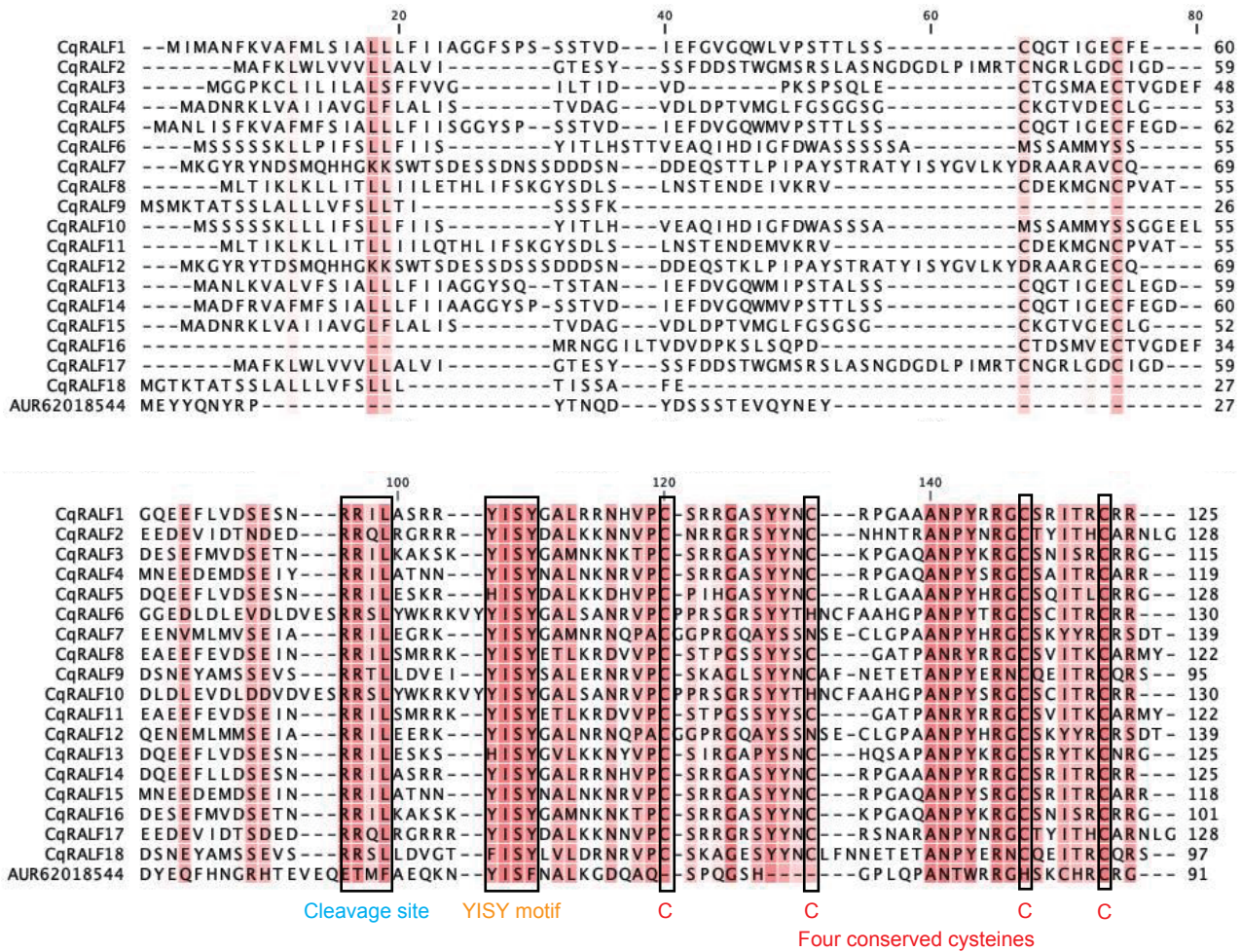

Supplement: Supplementary Figure 2 — Protein structural analysis of CqRALF peptides. The full-length amino acids of all 18 CqRALF peptides were aligned, and the motifs RRXL, YISY, and four conserved cysteines are indicated. [file Data_Sheet_2.PDF]
